# National laboratory policies and plans in sub-Saharan African countries: gaps and opportunities

**DOI:** 10.4102/ajlm.v6i1.578

**Published:** 2017-07-31

**Authors:** Pascale Ondoa, Ankie van der Broek, Christel Jansen, Hilde de Bruijn, Constance Schultsz

**Affiliations:** 1 Department of Global Health, Amsterdam Institute for Global Health and Development, Academic Medical Center, University of Amsterdam, Amsterdam, the Netherlands; 2 Royal Tropical Institute, Amsterdam, the Netherlands; 3 Ministry of Education, Culture and Science, International Policy Unit, The Hague, the Netherlands

## Abstract

**Background:**

The 2008 Maputo Declaration calls for the development of dedicated national laboratory policies and strategic plans supporting the enhancement of laboratory services in response to the long-lasting relegation of medical laboratory systems in sub-Saharan Africa.

**Objectives:**

This study describes the extent to which laboratories are addressed in the national health policies and plans created directly following the 2008 momentum for laboratory strengthening.

**Method:**

National health policies and plans from 39 sub-Saharan African countries, valid throughout and beyond 31 December 2010 were collected in March 2012 and analysed during 2013.

**Results:**

Laboratories were addressed by all countries. Human resources were the most addressed topic (38/39) and finances and budget were the least addressed (< 5/39). Countries lagging behind in national laboratory strategic planning at the end of 2013 (17/39) were more likely to be francophone countries located in West-Central Africa (13/17) and have historically low HIV prevalence. The most common gaps anticipated to compromise the implementation of the policies and plans were the disconnect between policies and plans, under-developed finance sections and monitoring and evaluating frameworks, absence of points of reference to define gaps and shortages, and inappropriate governance structure.

**Conclusion:**

The availability of laboratory policy and plan implementation can be improved by strictly applying a more standardised methodology for policy development, using harmonised norms to set targets for improvement and intensifying the establishment of directorates of laboratory services directly under the authority of Ministries of Health. Horizontal programmes such as the Global Health Security Agenda could provide the necessary impulse to take the least advanced countries on board.

## Introduction

Laboratory services are key to the quality of healthcare but have remained a historically-neglected component of health systems in low- and middle-income countries. The need for quality medical laboratory services to form an integrated part of the health system has been widely acknowledged by key national and international players during the past decade, resulting in the mobilisation of substantial funding earmarked for laboratory improvement in resource-limited settings.^1,2^

Alongside these efforts, the development of a national laboratory policy and strategic plan (NLSP) relevant to each country’s needs, and aligned with its health policy(ies) and plan(s), has also been advocated.^3,4,5^ A national laboratory policy defines the vision and the mission of a country’s laboratory system, whereas a strategic plan provides the corresponding roadmap guiding the process of the practical implementation of the necessary laboratory system improvement. The coherence of NLSPs with other national health guidance documents, such as national health policies, plans for development of human resources for the health sector, or disease-specific policies or plans, increases the likelihood that laboratory development strategies will be implemented.

Despite the recent prioritisation of laboratory services in global and national health agendas, various aspects of the laboratory system fail to meet standards in several countries of sub-Saharan Africa. Clinical diagnostic capacity often remains insufficient for the control of HIV, malaria, tuberculosis and other infectious diseases and for responding to the rise in incidence of non-communicable diseases.^6,7,8^ In addition, the lack of laboratory-based surveillance precludes the timely identification of emerging infectious disease threats.^9,10^ The recent Ebola virus disease epidemics^11^ and the paucity of systematic data on the antimicrobial resistance^12,13^ of key bacterial pathogens are two dramatic examples illustrating the lack of laboratory capacity to either respond to or anticipate public health events of national or international concern. The persistent shortage of laboratory workers in many countries of sub-Saharan Africa^14^ also highlights that crucial needs are still unmet.

The primary aim of this study was to describe to what extent laboratory services were addressed in the round of national health policies and strategies created by sub-Saharan African countries following the 2008 Maputo Declaration.^5^ The profile of countries lagging behind the process of laboratory strategic planning was explored.

## Methods

We conducted a desk review of national health policy documents. Eligible documents had to be valid throughout and beyond 31 December 2010 and were collected in March 2012. No updated or new policies were added after document collection was closed. The analysis was completed at the end of 2013.

### Selection of countries and documents

Sub-Saharan African countries identified by the World Health Organization as experiencing a crisis in human resources for health^15^ or receiving support from the United States President’s Emergency Plan for AIDS Relief (PEPFAR) were included in the study,^16^ which led to a review of 41 countries in total.

A search was carried out, per country for the following documents:

- National health strategy (or policy in case a strategy was not found).
- National human resources for health policy and/or strategy.
- National laboratory policy and/or strategy and/or operational plan.
- National HIV policy and/or strategy.
- National tuberculosis policy and/or strategy.
- National malaria policy and/or strategy.

Searches were carried out on the websites of the Ministries of Health, thematic websites (such as www.aidstar-one.com and the Roll Back Malaria website http://www.rbm.who.int/countryaction/index.html), Google, and within the networks of the Fondation Mérieux, the Association of Public Health Laboratories, the Royal Tropical Institute of the Kingdom of the Netherlands, and the Amsterdam Institute for Global Health and Development. Details on the search strategies and the web sites visited are provided in Supplementary Document 1.

### Additional information

Data on the political, economic and health status of each country were retrieved from the World Bank (www.worldbank.org), World Health Organization^17^ and UNAIDS^18^ databases. Information on PEPFAR collaboration, the existence of a department of laboratory services within the Ministry of Health and the degree of advancement of national laboratory strategic planning were retrieved from the PEPFAR (www.pepfar.gov) and World Health Organization databases (http://www.who.int), Ministry of Health websites and through direct questioning of in-country laboratory stakeholders.

### Document review

A data collection form was developed (Supplementary Document 2) and translated into a list of 88 codes pertaining to various aspects of national laboratory policy and strategic planning (Supplementary Document 3). The codes were grouped in broader families of codes, representing specific themes, e.g., ‘*attention to the public and the private sector*’, and according to a set of questions guiding the analysis. e.g., ‘*to which aspects of the laboratory do the policy and plans pay attention?’.* The coding framework was applied to all the documents available for review. A core team of two members searched the documents, entered the data in the form and discussed and reviewed each other’s work to reduce the risk of differences in interpretation of documents and coding of data. Following the review, and whenever necessary, the documents were screened for specific key words to ensure that all relevant sections had been properly coded and to allow description of content insufficiently covered by the coding list. Data with assigned meanings in Atlas.ti (Atlas.ti, version 6.2; ATLAS.ti Scientific Software Development GmbH, Berlin, Germany) were transferred to SPSS (IBM SPSS Statistics, version 20 for Windows; IBM Corp., Armonk, New York, United States).

### Analysis

For each country, we evaluated the compliance of policies and plans with guidance documents for laboratory policy and strategic planning,^19^ and the availability of concurrent and aligned national laboratory policies, strategies and operational plans according to the planning cycle. We examined the degree of harmonisation between policies and plans addressing the laboratory, the overall health sector and the development of human resources for health for each country.

Countries were categorised into groups of ‘*not started*’ versus ‘*started*’ with respect to the development and implementation of their NLSP at the time of the analysis in 2013 (Supplementary Table 1). These groups of countries were compared for proportions of low-, middle- and high-income countries or against their average HIV prevalence using the chi-square or Student’s *t*-test.

**TABLE 1:** Cross references between national laboratory, national human resources for health and national health policies or plans.

Countries with a laboratory policy/plan	Reference to health policy/plan within the laboratory policy/plan	Reference to the human resources for health policy/plan
Tanzania	Health Sector Strategic Plan III from 2009–2015	No
Malawi	Ministry of Health strategic plan 2007–2010 (previous to current one)	HRH strategic plan (not available for the analysis)
Rwanda	No	No
Zimbabwe	National Health Policy 2008–2012 (previous to current one)	No
Sierra Leone	Health Policy 2002 and Health Strategic Plan (no specific year)	No
South Africa	African National Congress National Health Plan 1994 (previous to current one)	National Human Resource Plan for Health 2006 (previous to current one)
Mozambique	Health Sector Strategic Plan (no specific year)	No
Ethiopia	Health Sector Development Programme	No (no plan found for analysis)
Democratic Republic of Congo	*Politique Nationale de Santé and Plan National de Développement Sanitaire* with details on strategies and policy elements that are integrated in the laboratory plan (2011–2015).	*Plan National de développement des ressources humaines pour la santé (2011–2015)*
Uganda	National Health policy and National Health Strategic Plan (no specific year)	HRH plan (no specific year)

HRH, human resources for health

The analysis of the coded data focused on the identification of gaps and opportunities in selected areas of the laboratory system: governance, private sectors, quality and accreditation, human resources, finances and monitoring and evaluation.

Attention to laboratory services was defined by the number of times that the laboratory was specifically addressed, and was quantified by calculating the frequency of each code or group of codes per document type, country or group of countries. Chronological alignment between policies and plans was evaluated by plotting inception and expiry dates of the documents against each other for each country.

## Results

**FIGURE 1:**
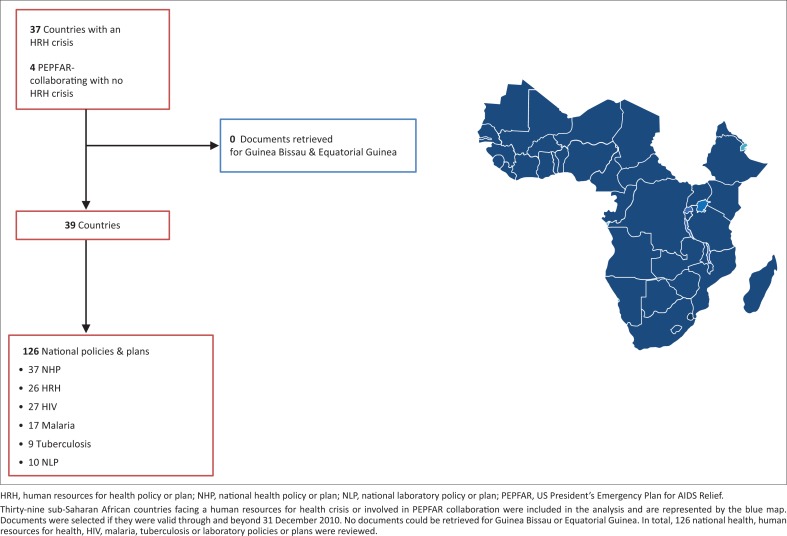
Countries and documents included in the study.

In total, 126 documents from 39 countries were found for review (Figure 1).

### Availability of aligned national laboratory policies, strategies and operational plans

Of the 10 countries (25.6%) with a national laboratory policy, strategy, or operational plan available for review, one had developed a combined policy and strategic plan within a single document and nine had developed either a laboratory policy or a laboratory strategic plan.

**FIGURE 2:**
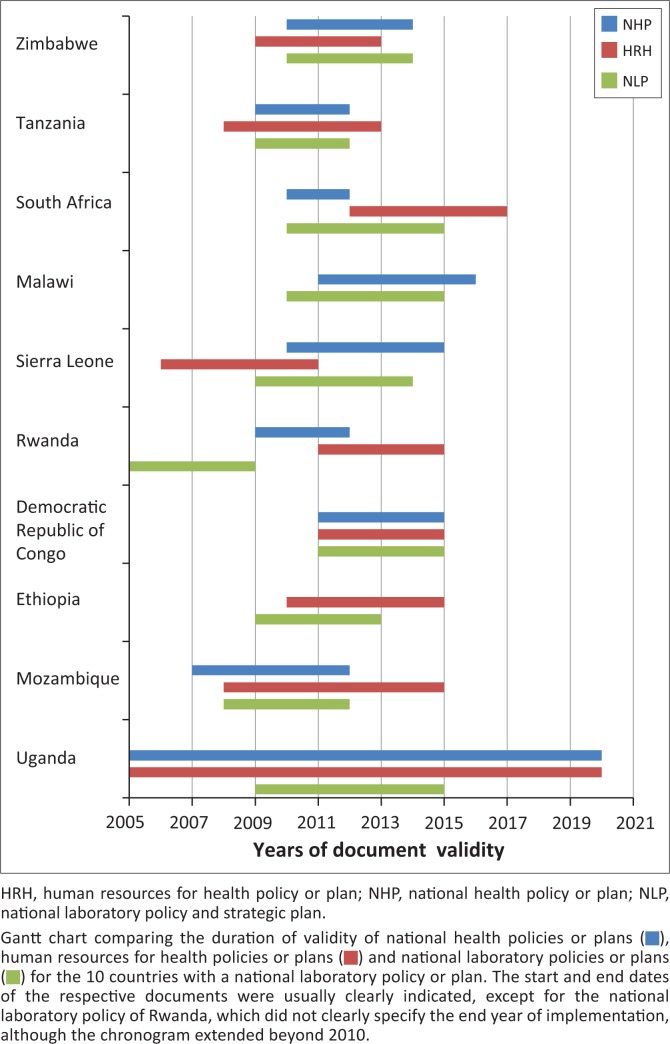
Overlap of validity time frames in 10 countries with a laboratory policy or plan available for review.

The start and end years of the national laboratory policies, strategies or operational plans; national health policies; and national human resources for health policies or strategies of the 10 countries with a laboratory document available for review are depicted in Figure 2. Lack of chronological overlap was identified between some policies and plans. In South Africa, for instance, the foreseen implementation time frame of the human resources policy lay largely outside the foreseen implementation timeline of the national health policy. In Sierra Leone, the human resources policy had become obsolete shortly after national laboratory policy implementation started. In Rwanda, the timeline of the national policies for laboratory, for human resources, and for health were not aligned. In contrast, the Democratic Republic of Congo had perfectly coordinated the timelines of all three national policies.

The 10 national laboratory policies were screened for any reference to either the national health policy or the human resources for health plan (Table 1). Nine of 10 countries referred to their national health policy in their national laboratory plan. Six of the 10 national laboratory policy documents did not refer to human resources for health plans.

### Factors associated with advancement of national laboratory policy and strategic planning in sub-Saharan Africa

Information gathered through the Association of Public Health Laboratories, PEPFAR, Fondation Mérieux, The Royal Tropical Institute and the Amsterdam Institute of Global Health and Development networks, indicated that 17 countries were not yet engaged in initial discussions regarding national laboratory strategic planning and were categorized as ‘*not started*’. The 23 other countries (of which 10 had a national laboratory policy available for review) were found to be either engaged in discussions, have developed a first draft, or (for the most advanced) have implemented, reviewed and evaluated their NLSP. These countries were classified as ‘*started*’. An update of the situation in early 2017 indicated that six additional countries are now categorised as ‘*started*’, making for a total of 29 (Supplementary Table 1).

**Supplementary Figure 1:**
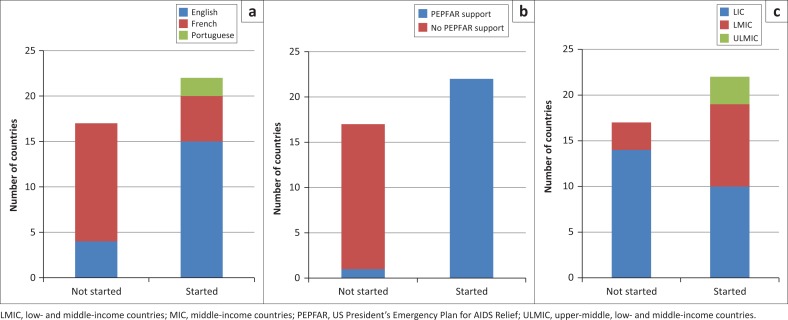
Comparison of groups of countries that have started versus not started the process of implementing a national laboratory policy and strategic plan.

Countries that had not yet started with their NLSP were more likely to be French-speaking, be classified as lower income, or not be receiving PEPFAR support compared to countries that had already started with the process of strategic planning (Supplementary Figure 1a and 1b). Countries categorised as ‘*not started*’ were also those with a lower HIV prevalence as compared to countries categorised as ‘*started*’ (average prevalence = 1.9% versus 9.5%, *p* = 0.001). There were no differences between the two groups of countries in terms of gross domestic product, percentage of gross domestic product spent on health, health expenses per capita, and percentage of external resources for health (Supplementary Figure 1c).

### Main laboratory themes addressed in health policies and plans

**FIGURE 3:**
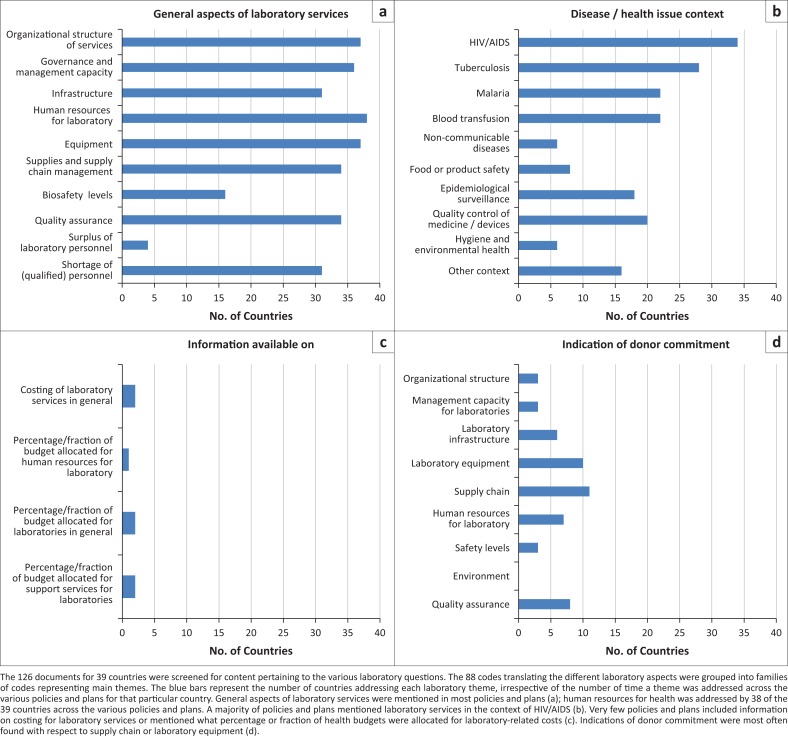
Attention to laboratory aspects in country policies and plans (N = 39).

The most common component of laboratory services addressed across various health policies (38/39 countries) was human resources for laboratory, mostly in terms of shortages (32/39) (Figure 3). All national laboratory and tuberculosis policies, strategies, and operational plans mentioned the issue of human resources for laboratory as compared with 25% of national health policies, 24% of the human resources for health policies, 19% of HIV policies, and 15% of malaria policies (data not shown).

Other main cross-cutting components of laboratory strengthening were mentioned as follows: governance (35/39); organizational structure and management capacity (36/39); laboratory infrastructure, equipment and supplies (36/39); and quality assurance (34/39), all issues were predominantly mentioned in the specific context of HIV/AIDS (34/39).

Biosafety was the least addressed cross-cutting component of laboratory services, being overlooked by 21 of the 39 countries (53.8%). Coverage of the eight main cross-cutting components of laboratory strengthening^19^ was achieved in the 10 countries with a national laboratory policy. Among the 29 countries without a dedicated laboratory policy or plan, four addressed all core aspects of laboratory strengthening through other type(s) of plans, while the majority overlooked one (*n* = 15) or several (*n* = 10) of the eight cross-cutting components concerning medical laboratories.

Budget allocation and donor support for laboratory services were scarcely covered (Figure 3), regardless the type of document studied. The percentage of health budgets earmarked for laboratory services or human resources was mentioned by only two countries. Among the 39 countries, donor support was hardly addressed in the context of the organisational structure of laboratories (*n* = 2), biosafety (*n* = 2), or management capacity (*n* = 4), and more frequently in the context of laboratory equipment (*n* = 10) and supply chain (*n* = 11).

### Most significant weaknesses identified in policies and plans

#### Little attention to financing

Regardless of the type of plan, very limited attention was devoted to the budget available or needed for the provision of laboratory services or for the implementation of laboratory improvement strategies. Evidence for a budget line dedicated to laboratory services was found in two out of the 39 countries. Of the 37 national health plans, three reported a specific part of the health budget allocated to support services and one indicated budget allocation for laboratory staff in general. Eleven countries provided a budget dedicated to specific strategies to improve laboratory services in their national health plans.

All 10 national laboratory policies reported insufficient government funding for laboratory services. At the same time, seven of these did not provide the percentage of the overall government health budget available for laboratory services. An overview of donor contributions was also lacking in all of the documents reviewed. Information on costing, budgeting and identification of funding sources for the implementation of the overall as well as specific components of the NLSP were largely missing. Funding from external partners was often mentioned in the context of the provision of equipment and supplies, or in relation to specific diseases, but rarely in the area of human resources development.

#### Lack of practical framework to monitor and evaluate laboratory services and national laboratory policy implementation

No indicators were proposed to measure the performance of laboratory services in any of the national laboratory policies. A monitoring and evaluation section to follow the implementation of the plan was included in four of the seven NLSP (Democratic Republic of Congo, Malawi, Tanzania and Zimbabwe), with only one (Democratic Republic of Congo) providing sufficiently detailed indicators linked to a budget and a chronogram.

#### Insufficient attention to the creation of laboratory departments within Ministries of Health

Only five countries described streamlining the administration of their laboratory systems through a laboratory directorate falling directly under the authority of the Ministry of Health, as recommended by the Maputo Declaration (Table 2). The other countries organised their laboratory services through a department responsible for, but not dedicated to, laboratories (*n* = 13), through a single service provider or research institution (*n* = 4), or through multiple entities within the Ministry of Health and provider institutions (*n* = 7). For 10 countries, no information on the laboratory coordinating body could be found.

**TABLE 2:** Type of laboratory governance in the 39 sub-Saharan African countries studied.

Dedicated Laboratory Directorate in Ministry of Health (*n* = 5)	Department responsible for laboratories and other sectors (e.g., Pharmacy) (*n* = 13)	Provider/research institution responsible for laboratory (*n* = 4)	Multiple departments within Ministry of Health dedicated to laboratory (*n* = 7)	Unknown (*n* = 10)
Burkina Faso	Benin	Ethiopia (EHNRI)	Uganda	Botswana
Senegal	Burundi	Rwanda (NRL)	Mozambique	Comores
Sierra Leone	Central African Republic	South Africa (NHLS)	Zambia	Congo
Zimbabwe	Chad	Mali (INRSP)	Namibia	Ghana
Democratic Republic of Congo	Côte d’Ivoire		Swaziland	Eritrea
	Gambia		Kenya	S. Sudan
	Guinea		Liberia	Angola
	Mauritania			Lesotho
	Niger			Nigeria
	Togo			Malawi
	Cameroon			
	Madagascar			
	Tanzania			

EHNRI, Ethiopian Health National Research Institute; INRSP, Institut National de Recherche en Santé Publique; NHLS, National Health Laboratory System; NRL, National Reference Laboratory

Of the seven national laboratory policies of countries with no directorate of laboratories, only two national laboratory policies explicitly aimed at raising the profile of laboratory services through one department within the Ministry of Health. Nineteen of the 21 national health policies (90.5%) addressing laboratory governance were from countries that did not yet have a directorate of laboratory services under the Ministry of Health. Of these, only two proposed the creation of a directorate of laboratories within the Ministry of Health as a way to improve the governance of laboratory services.

#### Poorly-informed situation analysis of the private laboratory sector

Private laboratories were addressed by 28 of 39 countries (71%) and by all national laboratory policies (data not shown). Five national laboratory policies described private laboratories as being separate from the public laboratory network. Six national laboratory policies, two human resources for health plans, one HIV policy and one malaria policy from eight of the 39 countries (84%) provided some information on the number of facilities operating in the private sector or the percentage of the population they serve or the laboratory staff they employ. The governance of the private sector and private sector links with the national laboratory network were left largely unclear in most of the policies and plans reviewed. Only three national laboratory policies provided some indication that the private sector was functioning as part of the overall laboratory network.

The list of participating stakeholders and summaries of meeting proceedings attached to the policies and plans suggested that the private laboratory sector and economic actors susceptible to support the implementation of the plan were only marginally involved in the development of the laboratory documents.

#### Insufficient points of reference for establishing workforce shortage reduction strategies

Twenty-six countries reported laboratory workforce shortages in their national health policies and/or human resources for health policies, four mentioned overstaffing and nine did not explicitly discuss shortages in the laboratory workforce. Among the countries indicating shortages, six did not provide any information on the category of laboratory workers concerned, 10 did not specify a point of reference to define the shortage and 20 did not have any information on the attrition rate of the laboratory workforce (Supplementary Table 2). Three countries defined shortages against positions actually available (vacancies), three against current or projected workload, and 11 against national norms based on tier-specific staffing requirements.

**Supplementary Figure 2:**
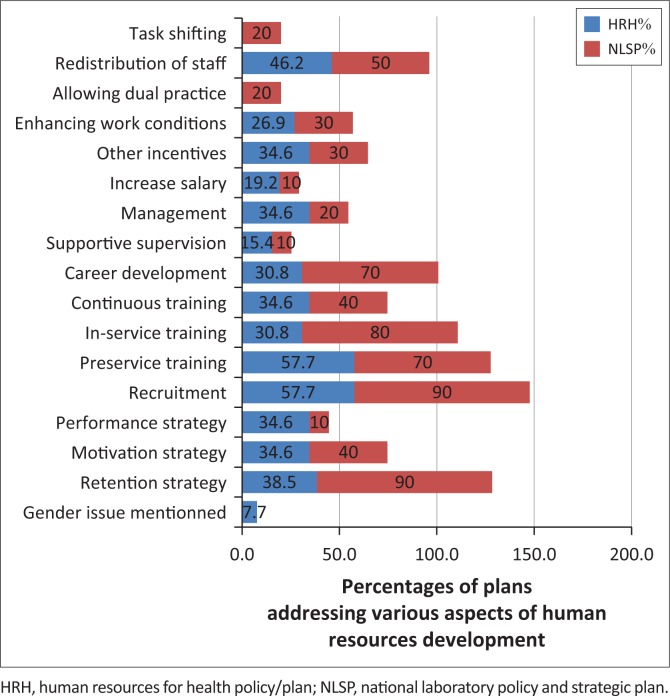
Aspects of human resources development addressed in 10 NLSP and 25 HRH.

National laboratory policies generally included more aspects of human resources for laboratory in their plans as compared to any other type of policy and plan (Supplementary Figure 2). However, eight of 10 NLP did not provide data on the categories of staff affected nor on points of reference to define the shortages. Clear strategies, targets and funding sources to improve the availability, capacity and performance of the workforce were not specified.

#### Vision and plan for laboratory accreditation not referred to in national laboratory policies and plans

Nine of the 10 national laboratory policies planned to use laboratory accreditation as a way to promote quality laboratory services, in line with the recommendations of the Maputo Declaration. However, roadmaps explaining how countries intend to move toward accreditation across diseases, at different tiers of the laboratory system and given the resources available were rarely provided or referred to (Box 1). Ethiopia was an exception, detailing an initial achievement of disease-specific international accreditation of polio and HIV reference laboratories, followed by a progressive expansion to other diseases and down to the regional level. Certification of laboratories based on compliance to national standards was never mentioned as a strategy to ensure the quality of laboratory services at lower tiers.

BOX 1 Strategies for laboratory accreditation described in the 10 national laboratory policies and plans.

- Ethiopia, Rwanda and Malawi plan to achieve international accreditation for reference (public) laboratories.
- Uganda already counts a few accredited laboratories and plans to move accreditation forward at all tiers for the public and private sector.
- In Sierra Leone, accreditation is foreseen to be a requirement for the licensing of all laboratories. Tanzania plans accreditation of laboratories without details on the tiers concerned, implying that both public and private laboratories might be involved.
- The Democratic Republic of Congo plans to accompany (public) laboratories to accreditation with no further details.
- South Africa plans to increase the number of accredited laboratories, especially in the public sector.
- Zimbabwe plans to strengthen the capacity of laboratories to be accredited in accordance with ISO 15189 by forming an accreditation body.
- Mozambique has plans to expand external quality assurance to several diseases at several tiers of the laboratory system, but does not mention accreditation of laboratories.

## Discussion

The attention devoted to medical laboratories in general, and to human resources in particular, in the round of national health policies and strategic plans that appeared shortly after the international momentum for the strengthening of laboratory services underscores the robust uptake of the recommendations of the Maputo Declaration in the majority of sub-Saharan African countries. However, 17 countries (43%) were still lagging behind the process at the time of the analysis in 2013. Strikingly, countries with less advanced laboratory policies and plans were mainly located in West and Central francophone Africa with a history of low HIV prevalence and limited PEPFAR investment. Combined with the observation that most laboratory issues were addressed in the context of HIV/AIDS, this finding illustrates the key role that international, vertical, disease-specific programmes, such as PEPFAR and the Global Fund, have played in prioritising and funding the development of laboratory systems in the region. At the same time, the report suggests that areas with smaller HIV epidemics benefited less from available opportunities to advance their national laboratory policy and strategic planning.

More equitable access to technical and financial resources to advance laboratory systems through adequate policy and planning could be achieved by diagonalising vertical and horizontal programmes in such a way that disease-wide, multi-sectoral and whole-of-government programmes for health improvement include the improvement of laboratory systems for the control of specific diseases. The Global Health Security Agenda goal to accelerate the achievement of International Health Regulations targets could offer a momentum for this paradigm shift, by incorporating laboratory-specific targets in the effort to achieve global health security.^20^

This report identifies several inadequacies which should be addressed in the upcoming round of NLSP to increase the likelihood of implementation of laboratory policies and plans and support the achievement of national and global health targets.

### Recommendations

#### Application of standardised methodologies

Consistently apply existing standardised methodologies for the development of national laboratory policies. This would ensure that no policy is formulated without a strategic plan or vice versa and that national laboratory policies are adequately aligned with other related health policies and integrate all key elements necessary for implementation. In addition, a (self-applied) national laboratory system assessment using available standardised tools, for example, the World Health Organization Laboratory Assessment Tool (LAT)-system assessment,^21^ Laboratory Network (LABNET) scorecard,^22^ and a strengths, weaknesses, opportunities, threats (SWOT) analysis of laboratory cross-cutting areas by a large inter-sectoral group of laboratory stakeholders conducted prior to the policy formulation, could facilitate the adequate prioritisation of strategic areas for improvements. Such a strictly standardised approach is ongoing for the development of national laboratory policies in Eastern Europe and central Asia, under the ‘Better Lab for Better Health Initiative’^23^ of the World Health Organization’s Regional Office for Europe, with promising results.

#### Harmonised national norms and standards

Define and disseminate harmonised national norms and standards for various aspects of laboratories as a priority for international partners in laboratory development. Regional bodies, such as the African Society for Laboratory Medicine or the World Health Organization’s Regional Office for Africa, could facilitate the development or the sharing and adjustment of national norms and standards available (e.g., staffing norms based on anticipated workload defined in Botswana; norms for infrastructure and equipment) from countries in similar settings. International technical partners could coordinate the development of generic costing tools for key items such as the implementation of quality management systems or laboratory information management systems or sample referral systems.

#### Renewal of advocacy efforts

Renew advocacy efforts for the creation of a directorate of laboratory services directly under the authority of the Ministry of Health, as recommended by the first call of the Maputo Declaration. With the correct mandate, a directorate can leverage improvements in several cross-cutting areas of laboratory systems such as development and enforcement of regulations, legislation (including the private sector), and development and application of normative standards (e.g., staffing, quality, infrastructure). The directorate would delineate important roadmaps such as national laboratory accreditation plans with linkage to laboratory-dedicated budget lines. Regional bodies and international development partners can significantly contribute to advocacy efforts, as illustrated by the RESAOLAB (*Réseau d’Afrique de l’Ouest des Laboratoires*) programme from Fondation Mérieux, which promoted the creation of laboratory directorates in Burkina Faso and Senegal.^24^

### Conclusions

The conclusions and recommendations stated here are mainly based on the analysis of documents that were available for review. The authors acknowledge the possibility that the outcome of the review could have been different, if all national policies had been available for the analysis. Additionally, the documents available from public sources may have been short versions that lacked budget sections. Finally, the present situation of national laboratory policy and strategic planning is different from that in 2010, with West and Central francophone African countries such as Mali, Burkina Faso, Burundi and the Republic of Congo currently implementing their first NLSP. In addition, six countries on the continent are implementing their second round of NLSP. Moving forward, the lessons reported here can be taken on board when countries develop or revise their laboratory policies, or initiate the next round of NLSP.

## Figures and Tables

**Table:** 

Label	Title
Supplementary Document 1	Search strategy
Supplementary Document 2	Analysis template
Supplementary Document 3	Code list
Supplementary Table 1	Status of national laboratory strategic plans in sub-Saharan African countries in 2013 and 2017
Supplementary Table 2	Evidence for laboratory workforce shortage and attrition provided in national policies or plans
Supplementary Figure 1	Comparison of groups of countries that had started versus not started the process of national laboratory strategic planning
Supplementary Figure 2	Aspects of human resource development addressed in 10 national laboratory policies and 25 human resources for health policies

**Table:** 

Countries	
Angola	Malawi
Benin	Mali
Botswana	Mauritania
Burkina Faso	Mozambique
Burundi	Namibia
Cameroon	Niger
Central African Republic	Nigeria
Chad	North Sudan
Comoros	Rwanda
Congo	Senegal
Cote d’Ivoire	Sierra Leone
DRC	South Africa
Equatorial Guinea	South Sudan
Eritrea	Swaziland
Ethiopia	United Republic of Tanzania
Ghana	The Gambia
Guinea	Togo
Guinea-Bissau	Uganda
Kenya	Zambia
Lesotho	Zimbabwe
Liberia	
Madagascar	

Period of the search: 13–27 March 2012.

**Table:** 

a: Human Resources for Health:	yes/no
b: diagnostic services including the lab services:	yes/no
c: a separate laboratory services:	yes/no

**Table:** 

National Health Policy:	yes/ no/ don’t know
National Health Strategic Plan:	yes/ no/ don’t know
Human Resources for Health Plan:	yes/ no/ don’t know
National laboratory plan:	yes/ no/ don’t know
Other:	[please provide]

**Table:** 

National Health Policy:	yes/ no/ don’t know
National Health Strategic Plan:	yes/ no/ don’t know
Human Resources for Health Plan:	yes/ no/ don’t know
National laboratory plan:	yes/ no/ don’t know
Other:	[please provide]

**Table:** 

Organisational structure of laboratory services (organogram):	yes/ no
Management capacity for laboratory services:	yes/ no
Laboratory infrastructure:	yes/ no
Laboratory equipment:	yes/ no
Supply chain (e.g. consumables):	yes/ no
Human resources for laboratory services:	yes/ no
Safety measurements:	yes/ no
Working environment:	yes/ no
Quality Assurance (e.g. accreditation/ standardisation of testing, supervision):	yes/ no

**Table:** 

Laboratory services in the context of HIV/AIDS:	yes/ no
Laboratory services in the context of Tuberculosis:	yes/ no
Laboratory services in the context of Malaria:	yes/ no
Blood transfusion services	yes/ no
Other:	[please provide]

**Table:** 

**Information on current staffing coverage (lab workers related to inhabitants) in the country?:**	Yes/ no/ not clear
**Information on the establishment for laboratory workers (the staff for which, in the public sector, a position and salary is reserved):**	Yes/ no/ not clear
**Information on targets for numbers of laboratory staff:**	Yes/ no/ not clear
**Based on workload studies:**	Yes/ no/ not clear
**Based on minimum packages of activities for health services:**	Yes/ no/ not clear
**International comparison:**	Yes/ no/ not clear
Other:	[please provide]
Source:	

**Table:** 

A: Medical laboratory scientist/medical technologist with university degree	(#available/ # needed)
b: Medical laboratory technicians	(#available/ # needed)
c: Laboratory assistants	(#available/ # needed)
d: Others	(#available/ # needed)
Source:	

**Table:** 

Pre-service (formal) education:	yes/ no/ not clear
Recruitment strategies:	yes/ no/ not clear
Tackling of gender inequality:	yes/ no/ not clear

**Table:** 

National Health Strategic Plan:	yes/ no/ don’t know
Human Resources for Health Plan:	yes/ no/ don’t know
National laboratory plan:	yes/ no/ don’t know
Other namely	

**Table:** 

Organizational structure of laboratory services (organogram):	yes/ no
Management capacity for laboratory services:	yes/ no
Laboratory infrastructure:	yes/ no
Laboratory equipment:	yes/ no
Supply chain (e.g. consumables):	yes/ no
Human resources for laboratory services:	yes/ no
Safety measurements:	yes/ no
Working environment:	yes/ no
Quality Assurance (e.g. accreditation/ standardisation of testing, supervision):	yes/ no
Blood transfusion services	yes/ no
Technical expertise	
Other:	[please provide]

**Supplementary Table 1:** Status of the national laboratory strategic plan of sub-Saharan African countries in 2013 and 2017.

Countries	Category	Comments on availability for review	NLSP status during the analysis in 2013	NLSP status in 2017
Botswana	Started	Not available for review	NLSP 1	NLSP 2
Eritrea	Not started	–	Not started with discussion	No further information
Ethiopia	Started	Available for review	NLSP 1	NLSP 2
Gambia	Not started	–	Not started with discussion	No further information
Ghana	Started	–	Discussion started	NLSP 1
Kenya	Started	–	NLSP 1 not valid beyond 2010. NLSP 2 in development	NLSP 2
Lesotho	Started	Not available	NLSP 1	NLSP 1
Liberia	Not started	–	Not started with discussion	Started with discussion
Malawi	Started	Available for review	NLSP 1	NLSP 1
Namibia	Started	Not available	NLSP 1	NLSP 1
Nigeria	Started	–	TWG formed	NLSP 1
South Africa	Started	Policy included in the review	Discussion started	TWG formed
Sierra Leone	Started	Available for review	NLSP 1	NLSP 2
South Soudan	Not started	–	Not started with discussion	No further information
Swaziland	Started	–	TWG formed	NLSP 1
Tanzania	Started	Available for review	NLSP 1	NLSP 2
Uganda	Started	Policy available for review	TWG formed	NLSP 2
Zambia	Started	–	TWG formed	NLSP 1
Zimbabwe	Started	Available for review	NLSP 1	NLSP 1
Angola	Started	–	Discussion started	NLSP 1
Benin	Not started	First policy expired in 2007	Not started with discussion	Not started with discussion
Burkina Faso	Not started	–	Not started with discussion	NLSP 1
Burundi	Not started	–	Not started with discussion	NLSP 1
Central African Republic	Not started	–	Not started with discussion	Not started with discussion
Cameroon	Started	–	Discussion started	NLSP 1
Chad	Not started	–	Not started with discussion	Not started with discussion
Côte d’Ivoire	Started	Not available for review	NLSP 1	NLSP 1
Comoros	Not started	–	Not started with discussion	No further information available
Republic of Congo	Not started	–	Not started with discussion	NLSP 1
Democratic Republic of Congo	Started	Available for review	NLSP 1	NLSP 1
Guinee Conakry	Not started	–	Not started with discussion	NLSP 1
Madagascar	Not started	–	Not started with discussion	No further information available
Mali	Started	–	Discussion started	NLSP 1
Mauritania	Not started	–	Not started with discussion	No further information available
Mozambique	Started	Available for review	NLSP 1	NLSP1
Niger	Not started	Policy validated in 2012 after the end of data collection	Not started with discussion	No further information available
Rwanda	Started	Available for review	NLSP1	NLSP 2
Senegal	Not started	–	Not started with discussion	TWG formed
Togo	Not started	First policy expired in 2006	Not started with discussion	Started discussion

NLSP, national laboratory policy and strategic plan; TWG, technical working group.

Seventeen countries are classified as ‘*not started*’ when they have *not started* the discussion for the development of their national laboratory strategic plans. All other countries are classified as ‘*started*’.

**Supplementary Table 2:** Evidence for laboratory workforce shortage and attrition provided in national policies and plans

Countries	Laboratory cadres for which a shortage exists	Points of reference for the indication of a shortage	Exact attrition rate of laboratory technicians known
Benin (HRH)	Engineer/ Higher technician/Technician category B/ Biomedical technician category B	60% gap when compared with the staffing norms	Annually 3% of lab and /or radiology technicians are projected to leave due to retirement.
Botswana (HRH)	Medical Laboratory Scientists (esp. senior)	National norms, vacancy levels, workload (for the National Drug Quality Control Laboratory).	No
Burkina Faso (HRH)	Laboratory technicians (TL) and Superior laboratory technicians (TSL)	Staffing norms	Biomedical diagnostic staff average attrition rate is 7% due to temporary unavailability, 19% due to resignation, 4% due to deaths, and 1% due to licensing issues.
Burundi (HRH)	Laboratory technicians	53% gap compared to norms	No
DRC	Not specified	Not specified	No
Ethiopia (NLP)	Laboratory personnel with specialised technical skills	Not specified	No
Ethiopia (NLSP)	Laboratory technicians	Projections for 2015 and 2020	No
The Gambia (HRH)	Laboratory technicians and laboratory technologists	Norms and vacancies	68% to the private sector, incl. NGOs, and abroad.
Ghana (HRH)	Laboratory technicians/ technologists	Norms, projections of staffing needs and training outputs	1.5% attrition (outward migration, deaths, resignations) is assumed in the projections
Guinea (NLSP)	Laboratory technicians	Norms	No
Kenya (HRH)	Laboratory technicians and technologists	6% compared to the approved establishment. Compared to the norms, an 85% gap of technologists and 37% gap for technicians is projected for 2012.	3% annual attrition is assumed for all cadres for the projections
Lesotho (HRH)	Laboratory technologists, laboratory technicians	Current supply and current minimum requirements, projected supply gap	2.8% annual turnover rate (loss to other occupations) is assumed for laboratory technologists. In the supply projections, an overall annual loss estimate of 7–7.6% is used for technologists, 8.2% for technicians.
Liberia (HRH)	Laboratory assistants, laboratory technicians, laboratory specialists	Staffing norms	No
Malawi	Not specified in any of NHP, NLSP, HRH, HIV	Not specified in any of NHP, NLSP, HRH, HIV	No
Mali (HRH)	Laboratory technicians and superior laboratory technicians	Not specified	No
Mozambique (NLP)	Medical Laboratory Technicians	Workload and distribution of available laboratory staff according to training level	No
Niger (HRH)	Laboratory technicians	When applying the medium/ high staffing norms	No
Rwanda (HRH)	Laboratory technicians	Staffing norms	No
Sierra Leone (NLP)	Laboratory technicians	National norms	No
South Africa (NLP)	Not specified	Not specified	No
South Sudan	Not specified in any of NHP, MAL, HIV	Not specified in any of NHP, MAL, HIV	No
Swaziland (NLSP)	Not specified	Not specified	No
Tanzania (NLP)	Laboratory and biomedical engineering staff, specialists in parasitology and histopathology.	Not specified	No
Togo	Not specified in any of NHP, HIV	Not specified in any of NHP, HIV	No
Uganda (NLP)	Highly qualified laboratory professionals	Not specified	No
Zimbabwe (NLP)	Laboratory scientists, specialists	59% vacancy rate for medical laboratory scientists / technologists compared with establishment	No

HRH, Human Resources for Health; MAL, national malaria policy or strategy; NHP, national health plan; NLP, national laboratory plan; NLSP, national laboratory policy and strategic plan.

## Supplementary Document 1: Search strategy

### Documents

National plans, strategies, costing frames

Valid (strategies and frameworks) or published (evaluations and reports) after the expiry date of 31 December 2010

National Health Policy or Plan

National Health Strategy

National Human Resources for Health (HRH) strategy (or policy)

National Laboratory strategy (or policy)

National HIV strategy (or policy)

National tuberculosis strategy (or policy)

National malaria strategy (or policy)

Costing frameworks for the above strategies

Websites (all accessed April 2013)

http://www.who.int/countryfocus/cooperation_strategy/listofccs/en/index.html (for country cooperative strategies of World Health Organization)

http://www.erepublic.org/egovincountriesmmo/mali.html (to assess websites of all governments of countries)

http://www.globalhivmeinfo.org/SystemsManagement/Lists/Ministries%20of%20Health/AllItems.aspx (for all Ministries of Health)

http://www.kit.nl/smartsite.shtml?ch=FAB&id=29313&Part=Resources (for lots of general information on Human Resources)

http://www.kit.nl/smartsite.shtml?ch=FAB&id=32449 (Eldis Human Resources for Health Dossier)

http://www.kit.nl/smartsite.shtml?ch=FAB&id=32467 (HRH Global Resource Center: A global library of human resources for health)

http://www.kit.nl/smartsite.shtml?ch=FAB&id=32196 (Human Resources for Health)

http://www.kit.nl/smartsite.shtml?ch=FAB&id=32432 (World Health Organization Collaborating Centres Global Database. Collaborating Centres dealing with Human Resources for Health Development)

http://www.kit.nl/smartsite.shtml?ch=FAB&id=32194 (World Health Organization Human Resources for Health)

www.theglobalfund.org/ (per country proposals can be read)

Also visit the websites of international donor organisations and initiatives: USAID, FHI, Roll Back Malaria, UNAIDS, KNCV, MSH, Partners in health, PEPFAR, JICA, USAID, MSH, Institut Pasteur, Fondation Mérieux and IRD (Institut de Recherche pour le Développement).

If documents are not yet found, do a Google search for:

“Health National Plan” – country “Human Resources for Health” – country “Strategic Health Plan” – country “Health Policy” – country “Workload study health” –country “Health workers” – country “Health Evaluation” – country “Laboratory services” – country

## Supplementary Document 2: Analysis template

**Country name:**

**Documents reviewed:**

Give proper documentation details: Author, Publisher, Title, Date of publishing

**National Governmental Documents:**

**..**

**..**

**National non-Governmental Documents**

**..**

**..**

**Multilateral agencies**

**..**

**..**

**Bilateral agencies**

**..**

**..**

**Global Health Initiatives (GFATM, GAVI)**

**..**

**..**

**World Bank**

**..**

**..**

**Other**

**..**

**..**

**Is there evidence of existing documents on this subject that are not reviewed?**

**Yes/no**

**If yes: which documents**

**Websites reviewed:**

Mention link and date accessed

**General**

HIV/AIDS prevalence/ incidence:

according to UNAIDS

according to current National Strategic/Health Plan

Tuberculosis prevalence/ incidence:

according to STOP TB

according to current National Strategic/Health Plan

Malaria prevalence/ incidence:

according to Roll Back Malaria

according to current National Strategic/Health Plan

Country identified with a Human Resources for Health crisis? Yes/no

Low income country: yes/no

Middle income country: yes/no

National health budget:

Proportion of the health budget contributed by donors:

### A: Participation in development of plans:

***1: Out of the documentation is there evidence that the following stakeholders have been participating in the development of national plans?***

Representative of:

Government

Ministry of Health

Ministry of Education

Ministry of Finance

Ministry of Defence

Other

Public sector laboratory services

Private non-profit sector laboratory services (e.g., non-governmental organisations, faith-based organisations)

Private sector laboratory services

Professional Health Association for Microbiologists/ Medical doctors/ Laboratory staff

Research groups

Others:

Unknown

***2: Within the Ministry of Health is there a special department for:***

### B: National Health Policies and Plans:

***3: Which policies and plans exist in the country?***

***4: Which National Health Policies and Plans pay attention to laboratory services?***

***5: If yes to any of the above, what type of laboratories are mentioned?***

a: Public laboratory services?

a.1: Primary care level

a.2: Secondary care level

a.3: Tertiary care level

a.4: National Reference Laboratory

b: Private laboratory services?

b.1: Primary care level

b.2: Secondary care level

b.3: Tertiary care level

b.4: Other

***6: Is there attention for strengthening of the public private mix in laboratory services?***

yes/ no

Describe :

***7: Which aspects in respect to laboratory services are mentioned in the plan?*** (indicate in which plan information is found)

***8: Are the following laboratory services mentioned separately?:***

### C: Human Resources for laboratory services: *(for this section the documents of all stakeholders will be analysed)*

(indicate in which plan information is found)

***9: Is there evidence that there is a shortage on laboratory personnel?***

Yes/ no/ not clear

***10: If yes, on what kind of indicators/criteria is this evidence based?***

***11: What kind of laboratory staff is lacking?***

***12a: Are there strategies to decrease the shortage on HR for health laboratories mentioned in the documents?***

Yes/ No/ Not clear

***12b: Are these strategies accompanied by indicators***?

Yes/ No/ Not clear

***12c: Are these strategies accompanied by a budget?***

Yes/ No/ Not clear

***12d: Are responsibilities regarding the implementation of these strategies indicated?***

Yes/ No/ Not clear

***13: If strategies are mentioned, what kind of strategies are mentioned in the documents?***

***HR development***

***HR management***

Career development

In-service training

Retention strategies (salary and other financial incentives)

Allowing dual practice

Other incentives (e.g. housing, transport)

Staff distribution (urban/rural)

Task shifting

Other: [please provide]

***14: Does the plan provide investment in the training institutions for laboratory workers?***

yes/ no/ not clear

***15: If yes:***

a: Investment in the infrastructure (e.g. buildings, equipment)/ learning materials of these training institutions

b: Investment in lecturers/ tutors of these schools

c: Other

d: Not specified

***16: Does evidence exist about high attrition of lab personnel?***

Yes/ no/ not clear

***17 : If available, what is the attrition rate of laboratory staff?***

Rate…/ unknown

***18: If disaggregated, what is the attrition rate of:***

a: Medical laboratory scientist/medical technologist with university degree:

b: Medical laboratory technicians:

c; Laboratory assistants:

d: Others:

***19: Is there information on where laboratory staff migrate to?***

yes/ no/ not clear

***20: If yes, do they migrate***

a: from public health sector to private for profit health sector

b: from public health sector to international NGO/ agencies (health)

c: to the non-health laboratory sector

d: out of the country

e: from private non-profit sector to public sector

f: from private for-profit sector to public sector

g: other

### D: FINANCING FOR LABORATORIES

(indicate in which plan information is found)

***21: What is the total health budget for the country?***

a: Which proportion of the total government budget is earmarked for the health sector? - Budget:…

- Unknown

(source from: http://www.who.int/nha/country/en/index.html#U)

b: How much does the government spend on health per capita in US dollar terms?- Budget:…

- Unknown (same source as A)

c: Which proportion of the total health budget is planned/used for medical support services (Lab, pharmacy and radiology?)

- Budget:…

- Unknown

e: Which proportion of the total health budget is planned/used for laboratory services?

- Budget:…

- Unknown

f: Which proportion of the budget for laboratories is planned/used for HRH?

- Budget:…

- Unknown

Costing:

***22: Are laboratory services in the national health plans costed?***

***23: Are there national or international donors that invest in laboratory services?***

yes/ no/ not clear

Source:

***24: If yes, Which donors?***

***25: Which laboratory services are supported by these donors?***

***26: Do these donors invest in remuneration of lab staff (top up of salary/ incentives)?***

yes/ no/ not clear

**E: IMPLEMENTATION OF PLANS**

***27: Are there monitoring and evaluation reports or other documents that give evidence that laboratory components of plans implemented?***

a: evidence of implementation of laboratory components of NLSP

yes/ no/ not clear

b: evidence of implementation of laboratory components of donor plans

yes/ no/ not clear

***28: If yes: which elements of laboratory services are implemented?***

a: Describe the implemented laboratory components of NLSP:

b: Describe the implemented laboratory components of donor plans

## Supplementary Document 3: Code list

0. This relevant document was not found for review

1.0 Plan developed with multiple Ministry of Health departments

1.1 Plan developed with multiple ministries

1.2 Plan developed with public or private laboratory service providers

1.3 Plan developed with professional associations

1.4 Plan developed with researchers or universities

1.5 Plan developed with others

1.6 Unknown who participated in plan development

2.1 The Ministry of Health has a special HRH department

2.2 The Ministry of Health has a department that includes laboratory services

2.3 The Ministry of Health has a separate department for lab services

3.1.1 Public sector lab services mentioned in the plan

3.1.2 Private sector lab services mentioned in the plan

3.1.3 Attention for strengthening the public-private mix

3.2.1 Primary care level labs mentioned

3.2.2 Secondary level labs mentioned

3.2.3 Tertiary care level labs mentioned

3.2.4 National reference laboratory mentioned

4.1 Organisational structure of lab services mentioned in the plan

4.2 Governance and management capacity for lab services mentioned in the plan

4.3 Lab infrastructure mentioned in the plan

4.3.6 HRH for lab services mentioned in the plan

4.4 Laboratory equipment mentioned in the plan

4.5 Supplies and supply chain mentioned in the plan

4.6 Safety levels mentioned in the plan

4.8 Quality assurance of laboratory services mentioned in the plan

4.9 Other aspects of laboratory services mentioned in the plan

5.0.1 Laboratory services mentioned in the context of HIV/AIDS

5.0.2 Laboratory services mentioned in the context of Tuberculosis

5.0.3 Laboratory services mentioned in the context of malaria

5.0.4 Laboratory services mentioned in the context of blood transfusion services

5.0.5 Laboratory services mentioned in the context of non-communicable diseases

5.0.6 Laboratory services mentioned in the context of food or product safety

5.0.7 Laboratory services mentioned in the context of epidemiological surveillance

5.0.8 Laboratory services in the context of quality control of medicines/ medical devices

5.0.9 Laboratory services mentioned in the context of hygiene and environmental health

5.0.91 Laboratory services mentioned in other context

5.1.1 Different cadres mentioned, including laboratory personnel

5.1.2 Different cadres mentioned, excluding laboratory personnel

5.1.3 Different cadres mentioned, unclear whether laboratory personnel is included

6.0 There is a surplus of laboratory personnel

6.1 There is a shortage of (qualified) laboratory personnel

6.1.1 There is a shortage of medical laboratory scientists/ medical technologists with university degree

6.1.2 There is a shortage of laboratory technicians

6.1.3 There is a shortage of laboratory assistants

6.1.4 There is a shortage of other laboratory personnel

7.1 The evidence for the shortage of laboratory personnel is based on information on current staffing coverage (laboratory workers per no. inhabitants or per facility) in the country

7.2 The evidence for the shortage of laboratory personnel is based on available positions for laboratory personnel in the public sector

7.3 The evidence for a shortage of laboratory personnel is based on the mentioning of training needs

7.4 The evidence for the shortage of laboratory personnel is based on other targets/ sources

7.5 Unknown where the qualification ‘shortage’ is based on

8 Exact attrition rate of laboratory technicians

8.01 Strategy accompanied by indicator

8.02 Strategy accompanied by budget

8.03 Strategy accompanied by responsibilities

8.1.1 Gender issues related to HRH in laboratories

8.1.2 Retention strategy

8.1.3 Motivation strategy

8.1.4 Performance strategy

9.1 Pre-service (formal) education

9.2 Recruitment strategies

9.3 In-service training

9.4 Continuous professional development

9.5 Investment in career development of laboratory staff

9.6 Supportive supervision

9.7 Management

9.8 Increase salaries

9.9 Other incentives (housing, transport, non-financial)

9.91 Enhancing working conditions

9.92 Allowing dual practice

9.93 Urban-rural distribution of staff

9.94 Task shifting

9.95 Other

91.1.2 % of the health budget allocated to support services

91.1.3 % of the health budget allocated to laboratories

91.1.4 % of the health budget allocated to laboratory HRH

92.2.1 Laboratory services in general are costed

92.2.2 This specific laboratory service is costed

93.3.1 This plan shows evidence of a donor committing to invest in organisational structure

93.3.2 This plan shows evidence of a donor committing to invest in management capacity for laboratories

93.3.3 This plan shows evidence of a donor committing to invest in lab infrastructure

93.3.4 This plan shows evidence of a donor committing to invest in lab equipment

93.3.5 This plan shows evidence of a donor committing to invest in supply chain

93.3.6 This plan shows evidence of a donor committing to invest in HRH

93.3.7 This plan shows evidence of a donor committing to invest in safety levels

93.3.8 This plan shows evidence of a donor committing to invest in environment

93.3.9 This plan shows evidence of a donor committing to invest in quality assurance

93.3.91 This plan shows evidence of a donor committing to invest in other aspects of laboratory services
